# Caoutchouc Electuary

**Published:** 1873-11

**Authors:** 


Caoutchouc Electuary.—At the solicitation of several friends I have been induced to call the attention of the profession to the use of caoutchouc as a remedial agent. I have been in the habit for the past fifteen years of prescribing it, in preference to cod-liver oil, in certain cases of pulmonary tuberculosis in every stage, in chronic bronchitis, or the winter coughs of old people, and in chronic rheumatism.

Under its use I have observed a decided improvement in digestion and hæmatosis, as well as general tonic effects. It seems to be adapted to cases in which cod-liver oil is contra-indicated, and especially those in which there is a frequent occurrence of hæmoptysis. It is not claimed to be a remedy which has any specific action, but that it diminishes excessive mucous secretion and suppuration, arrests hemorrhage and excessive perspiration, and retards emaciation.

Prepared in the following manner, I have usually prescribed it in doses of a teaspoonful three times a day, about two hours *after* meals.

SOLUTION OF CAOUTCHOUC.

℞. Caoutchouc (in thin slices)............... ℥ i.

Ol. terebinth............................ ℥ ij.

M.

Macerate until solution is effected, and strain through coarse muslin.

ELECTUARY OF CAOUTCHOUC.

℞. Solut. Caoutchouc...................... ʒ ij.

Sacch. Alb.............................. ℥ iss.

Mellis (strained)....................... ℥ iiss.

M.

The mixture should be of opaque yellow color, and thick enough to run *very* slowly off a spoon. It contains about two grains of pure caoutchouc to each teaspoonful.

